# Innovation in Onychomycosis Treatment Through Creative Compounding

**DOI:** 10.7759/cureus.91475

**Published:** 2025-09-02

**Authors:** Kristine Hoffman, Matthew Gorski, Christian P Frey

**Affiliations:** 1 Orthopedics, Denver Health and Hospital Authority, Denver, USA; 2 Podiatry, Denver Health and Hospital Authority, Denver, USA

**Keywords:** compounded medication, nail lacquer, onychomycosis, terbinafine, topical antifungal therapy

## Abstract

Onychomycosis is a common fungal nail infection, often presenting with nail discoloration, thickening, and brittleness. Despite the availability of oral and topical antifungal agents, recurrence rates remain high, and systemic therapies can pose risks such as hepatotoxicity and drug interactions. This case report describes an innovative treatment approach using a compounded topical preparation made by combining a crushed 250 mg oral terbinafine tablet with an over-the-counter 1% tolnaftate nail lacquer. A healthy 28-year-old female presented with diffuse toenail onychomycosis affecting all digits. After declining oral therapy, she initiated daily application of the compounded lacquer for 10 months. Weekly complete removal and reapplication were performed, and the vial was shaken before each use to maintain a homogenous mixture. The patient documented progress photographically and reported high satisfaction with a significant improvement in nail appearance. No adverse effects were noted. This topical use of pulverized terbinafine takes advantage of the drug's favorable molecular profile for nail penetration, offering targeted treatment with minimal systemic absorption. The estimated daily delivery of terbinafine per nail was approximately 0.4 mg, a potentially therapeutic dose when applied consistently over time. This case supports the potential of creative compounding in expanding safe and patient-friendly treatment options for onychomycosis, especially for individuals who are unable or unwilling to take oral antifungals. Further studies are needed to evaluate the efficacy, pharmacokinetics, and broader applicability of this approach.

## Introduction

Onychomycosis is a highly prevalent fungal infection of the nail unit, caused by dermatophytes, non-dermatophyte molds, and yeasts. Clinically, it often presents with nail discoloration, thickening, and changes in consistency. It is estimated that onychomycosis accounts for at least 50% of all nail disorders worldwide [1-3]. Although it can affect both fingernails and toenails, toenails are approximately seven times more likely to be involved, likely due to localized trauma, the moist environment within shoe gear, and the inherently slower growth rate of toenails compared to fingernails [4]. These same factors contribute to the difficulty in eradicating the infection once fungal inoculation has occurred. Consequently, onychomycosis is a condition commonly managed by podiatrists, with many patients primarily seeking treatment for cosmetic concerns.

Over the years, various nail-sparing treatment modalities have been attempted, including nail soaks, mechanical debridement, topical antifungal lacquers, oral antifungal agents, laser therapy, and even non-permanent nail plate avulsions. While some success has been achieved, recurrence rates remain high, often leading to patient dissatisfaction and frustration.

Terbinafine is one of the most commonly prescribed oral antifungal medications for toenail onychomycosis, with reported success rates ranging from 35% to 78% [5-9]. As a squalene epoxidase inhibitor, terbinafine disrupts fungal cell membrane synthesis by inhibiting ergosterol production. However, its use is not without potential risks. Documented side effects range from the more common GI upsets and headaches to the less common visual or taste disturbances [10]. Hepatotoxicity, though rare, remains a significant concern due to hepatic metabolism of this medication. Additionally, clinicians must be cautious of potential drug-drug interactions with other hepatically metabolized medications. The standard dosing regimen is 250 mg orally once daily for 12 weeks, with baseline and periodic liver function monitoring strongly recommended [11].

In this case report, we describe an innovative approach utilizing a combination of an over-the-counter antifungal nail lacquer and a powdered prescription oral antifungal agent, offering a potentially safe, accessible, and effective alternative treatment option for onychomycosis in a healthy patient.

## Case presentation

The patient is a healthy 28-year-old female with a history of left foot bunion surgery in 2020 but no other significant pedal history. She reported progressive yellowing and thickening of all toenails, without any known inciting trauma. The patient admits to walking barefoot at yoga classes and occasionally in the locker room. Prior to presentation, she had attempted mechanical debridement at home without significant improvement.

Clinical examination revealed mild thickening and yellow discoloration involving all toenails. Subungual debris was noted at the distal one-third to one-half of the nail plates. The nail surfaces appeared brittle with irregular texture, and mild hyperkeratosis of the nail beds was observed. There was no surrounding erythema, edema, or other signs of acute infection. Based on the patient's history of participating in barefoot activity and physical examination, an empiric diagnosis of onychomycosis was made; no formal diagnostic testing was performed on the nail.

After a detailed discussion of the risks and benefits of various treatment options, a novel treatment strategy was initiated. The patient agreed to use an over-the-counter antifungal nail lacquer containing tolnaftate 1%, mixed with a pulverized 250 mg oral terbinafine tablet. The compounded lacquer was applied daily to each toenail, with complete removal and reapplication performed weekly. The patient was instructed to thoroughly shake the vial before each use to ensure a homogenous mixture.

**Figure 1:**
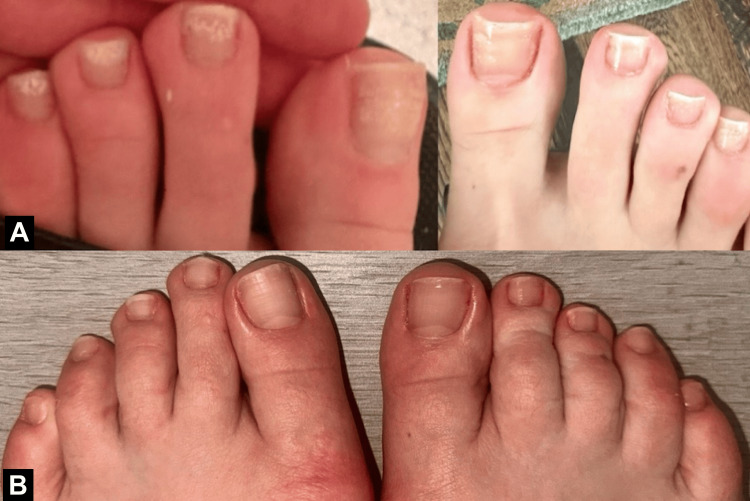
Before and after completing treatment Clinical photographs showing toenails before (A) and after (B) 10 months of daily application of a compounded topical antifungal lacquer containing tolnaftate 1% and pulverized terbinafine. A notable improvement is seen in nail clarity, thickness, and a reduction in discoloration and subungual debris.

Photographs were taken by the patient prior to treatment initiation and at regular intervals during the treatment course. Final before-and-after images are shown in Figure 1. The patient adhered closely to the application protocol over a period of 10 months. She reported a significant improvement in the appearance of her nails and expressed high satisfaction with the results. No adverse reactions or complications were reported during the course of treatment.

## Discussion

Onychomycosis remains a persistent challenge in podiatric medicine due to the unique structure of the nail plate and the difficulty of achieving effective antifungal penetration. Oral antifungal therapy, such as terbinafine, is often considered first-line treatment due to superior cure rates compared to topical therapies alone. However, concerns regarding systemic side effects, particularly hepatotoxicity, as well as potential drug-drug interactions, frequently limit its use, especially in patients seeking a non-systemic approach.

In this case, we explored an alternative method by repurposing oral terbinafine for topical administration in combination with a commercially available tolnaftate-containing lacquer. This compounded preparation aimed to enhance local drug delivery directly to the site of infection while minimizing systemic exposure and associated risks. The patient's high level of compliance, absence of adverse effects, and subjective improvement in nail appearance over a 10-month period suggest that this approach may offer a promising adjunct or alternative for select patients who are hesitant to pursue oral therapy or who have contraindications to systemic antifungals.

Several considerations supported the plausibility of this topical approach. Previous quantitative Structure-Property Relationship (QSPR) modeling and systematic reviews have shown that antifungal agents with low molecular weight and hydrophilic properties demonstrate improved nail penetration [12]. Nail permeability also depends on the chemical composition of the nail plate and keratinized tissue, both of which act as a barrier to diffusion [5,13]. The daily amount of terbinafine delivered topically via this compounded lacquer was approximately 0.4 mg per nail, which was therapeutic when considering diffusion dynamics over time and repeated application.

While terbinafine is traditionally administered orally, its physicochemical profile is not inherently unsuitable for topical delivery. Efinaconazole, a newer topical azole, contains a comparable active concentration of 10% in its solution and achieves measurable efficacy despite the challenge of nail barrier penetration [14-16]. This suggests that topical penetration of terbinafine in appropriate vehicles may yield clinically significant results. In fact, the principle is analogous to the SGLT2 inhibitor Jardiance, where systemic delivery mechanisms were successfully challenged and adapted for broader utility.

## Conclusions

The combination of a tolnaftate-based nail lacquer with pulverized terbinafine presents a potentially safe, effective, and patient-friendly alternative for managing onychomycosis. This novel strategy may be particularly valuable for patients who are unable or unwilling to undergo systemic antifungal therapy. Further investigation, through controlled studies evaluating different terbinafine concentrations, application frequencies, and larger case series, is warranted to more clearly define the role of compounded topical antifungal therapies within the treatment paradigm for onychomycosis.
